# Dual- versus single-agent HER2 inhibition and incidence of intracranial metastatic disease: a systematic review and meta-analysis

**DOI:** 10.1038/s41523-021-00220-0

**Published:** 2021-02-18

**Authors:** Anders Wilder Erickson, Steven Habbous, Christianne Hoey, Katarzyna J. Jerzak, Sunit Das

**Affiliations:** 1 Institute of Medical Science, University of Toronto, Toronto, Canada; 2 Ontario Health (Cancer Care Ontario), Toronto, ON Canada; 3 Evaluative Clinical Sciences, Sunnybrook Research Institute, Sunnybrook Health Sciences Centre, Toronto, Canada; 4 Department of Medicine, Sunnybrook Odette Cancer Centre, University of Toronto, Toronto, Canada; 5 Division of Neurosurgery, University of Toronto, Toronto, Canada; 6 Li Ka Shing Knowledge Institute, St. Michael’s Hospital, University of Toronto, Toronto, Canada

**Keywords:** Metastasis, Cancer epidemiology, Cancer, Breast cancer

## Abstract

Observational studies have suggested that HER2 inhibition with trastuzumab may be associated with an increased incidence of intracranial metastatic disease (IMD) due to its ability to prolong survival. We hypothesized that prolonged survival associated with dual-agent HER2 inhibition may be associated with an even higher incidence of IMD. This study pooled estimates of IMD incidence and survival among patients with HER2-positive breast cancer receiving dual- versus single-agent HER2 targeted therapy, as well as trastuzumab versus chemotherapy, observation, or another HER2-targeted agent. We searched PubMed, EMBASE, and CENTRAL from inception to 25 March 2020. We included randomized controlled trials that reported IMD incidence for patients with HER2-positive breast cancer receiving trastuzumab as the experimental or control arm irrespective of disease stage. Among 465 records identified, 19 randomized controlled trials (32,572 patients) were included. Meta-analysis of four studies showed that dual HER2-targeted therapy was associated with improved overall survival (HR 0.76; 95% CI, 0.66–0.87) and progression-free survival (HR 0.77; 95% CI, 0.68–0.87) compared to single HER2-targeted therapy, but the risk of IMD was similar (RR 1.03; 95% CI, 0.83–1.27). Our study challenges the hypothesis that prolonged survival afforded by improved extracranial disease control is associated with increased IMD incidence.

## Introduction

Intracranial metastatic disease (IMD) is a common and serious complication of breast cancer^1^, with a median survival of 13.8 months^2^ and reduced quality of life due to disease symptomatology and treatment toxicity^3^. Breast cancers expressing human epidermal growth factor receptor 2 (HER2) have a higher propensity to metastasize to the central nervous system (CNS) compared to hormone receptor (HR)-positive/HER2-negative disease subtypes^4,5^

The anti-HER2-monoclonal antibody trastuzumab has been shown to improve overall survival (OS) for HER2-positive breast cancer patients and has become standard of care^6,7^. However, case series and cohort studies have reported a higher incidence of IMD among HER2-positive patients treated with trastuzumab for metastatic, unresectable, or recurrent breast cancer^8–16^. Meta-analyses of randomized controlled trials (RCTs) have corroborated such findings in non-metastatic disease, but have not included data accrued in recent years^17–21^. The increased IMD incidence following treatment with trastuzumab has been attributed to its improvement of OS: trastuzumab controls extracranial disease and prolongs survival until dormant micrometastases within the sanctuary of the CNS are able to proliferate and manifest clinically ^22,23^.

The effectiveness of HER2 inhibition for HER2-positive breast cancer has motivated the development of novel HER2-targeted agents and trials to determine their efficacy as single agents or in combination with trastuzumab. RCTs have demonstrated the efficacy of several HER2-targeting agents, including anti-HER2 antibodies and conjugates (trastuzumab emtansine and pertuzumab) or HER2-targeted tyrosine kinase inhibitors (TKIs; lapatinib, neratinib, and tucatinib)^24–31^. Ongoing studies for margetuximab^32^, pyrotinib^33^, trastuzumab deruxtecan^34^, ARX788^35^, and PRS-343^36^ may expand upon or improve current options for patients with HER2-positive breast cancer, and illuminate the impact of additional HER2-targeted agents on IMD incidence.

HER2-targeted agents have also garnered interest in the treatment of IMD from HER2-positive breast cancer. Trastuzumab and other HER2-targeted antibodies and conjugates have been associated with a reduced number of intracranial tumors at IMD diagnosis, prolonged post-IMD OS, or intracranial responses, indicating possible intracranial efficacy despite minimal blood-brain barrier penetrance^37–39^. Recent reviews address the landscape of HER2-targeted agents for the management of IMD, and suggest that the introduction of novel agents, greater inclusion of patients with IMD in clinical trials, and increased reporting of intracranial outcomes may all benefit future patients with IMD^40,41^.

The purpose of this systematic review and meta-analysis was to update existing estimates of the incidence of IMD among patients with HER2+ breast cancer, and to assess the impact of novel HER2-targeted regimens on the development of intracranial metastases. To address this question, we assessed IMD incidence and survival among patients with HER2-positive breast cancer who were treated with dual anti-HER2 therapy versus trastuzumab monotherapy; we also performed meta-analysis of IMD incidence in patients receiving trastuzumab versus chemotherapy, observation, or another HER2-targeted agent.

## Methods

We followed the Preferred Reporting Items for Systematic Reviews and Meta-Analyses (PRISMA) guidelines^42^.

### Search strategy

We searched MEDLINE (via PubMed), EMBASE (via Wiley), and CENTRAL (via Cochrane) on March 25, 2020. We also screened references of eligible articles and reviews, and queried Google Scholar, PubMed, and ClinicalTrials.gov for updated or IMD-specific publications of trials at full-text review. Full search queries are presented in the supplement (Supplementary Tables 1–3).

### Study selection

Using a two-step process, we screened abstracts and then full texts of selected records to identify RCTs that reported the incidence of IMD that compared dual HER2-targeted regimens to trastuzumab, trastuzumab to another HER2-targeted therapy, or trastuzumab to standard chemotherapy or observation. Studies were screened in duplicate by two independent reviewers (AE, CH), and Cohen’s κ statistic was calculated for inter-rater reliability at both steps. Disagreements were resolved through discussion. Studies could report IMD incidence overall or as the site of the first recurrence. Trials that did not report IMD incidence were excluded. Gray literature sources were not searched. Conference abstracts were eligible. No date range was applied, but studies were required to be in English. Full inclusion and exclusion criteria are presented in the supplement (Tables S4–5).

### Data extraction and quality assessment

The following data were extracted from included studies: trial name, treatment procedures, median follow-up, prior treatments, early (stage I–II) versus advanced (stage III–IV) HER2-positive breast cancer, number of intracranial events, number of recurrence events, OS (hazard ratio) and progression-free survival (PFS, hazard ratio), reported either as disease-free, progression-free, or event-free survival. Outcomes specific to intention-to-treat analyses were preferentially extracted. Data were extracted by a single reviewer due to resource constraints. We performed a quality assessment using the Cochrane Risk of Bias 2 tool (RoB 2) to evaluate risk of bias across five domains (randomization, deviation from intended interventions, missing outcomes, measurement bias, and selection bias) and overall^43^. We assessed evidence quality using the Grading of Recommendations Assessment, Development, and Evaluation (GRADE) framework ^44^.

### Data synthesis and analysis

We performed a meta-analysis using the inverse variance method with random-effects models to produce summary risk ratios (RR) and hazard ratios (HR). We calculated RR using the number of cases of incident IMD divided by the number of patients in the intention-to-treat population per study arm. HR were extracted as adjusted HR when available, otherwise as reported. Missing HR values from one study^6^ were imputed using the method by Guyot et al.^45^ from digitized Kaplan–Meier plots and at-risk tables.

To assess heterogeneity, we calculated the Q-statistic for the ratio of observed to within-study variance, τ^2^ for between-study variance, and *I*^2^ for the percentage of observed variance attributable to between-study variance^46,47^.

We evaluated the incidence of IMD among patients receiving HER2-targeted therapy through two separate comparisons: dual HER2-targeted therapy versus trastuzumab; and trastuzumab versus chemotherapy, observation, or another anti-HER agent. Patients were pooled from treatment arms that received the same chemotherapy plus trastuzumab combination, but in concurrent or sequential order, or for different durations. As subgroup analyses, we estimated summary effects stratifying by disease stage, and in comparisons of trastuzumab monotherapy to control regimens, by the comparator. As sensitivity analyses, we compared summary estimates from fixed- and random-effects models, and omitted studies one at a time as a “leave-out-one” assessment^47,48^.

We assessed publication bias by examining the funnel plot asymmetry visually and with Egger’s test^49^. All statistical analysis was conducted using the R programming language (v3.6.1, R Foundation for Statistical Computing)^50^ and the R package meta^51^.

## Results

**Fig. 1:**
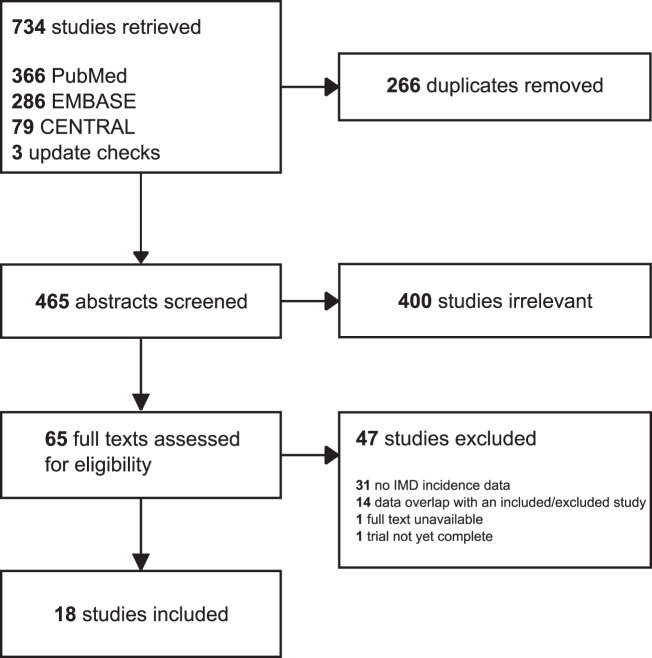
PRISMA flow diagram. Search queries were conducted in PubMed, EMBASE, and CENTRAL from their inception to 25 March 2020 for randomized controlled trials investigating trastuzumab that reported incidence of intracranial metastatic disease^42^.

Our search identified 465 unique records, from which we reviewed 65 full-text articles. Eighteen studies^6,25,28,29,52–65^ reporting on nineteen trials met eligibility criteria (Fig. 1); notably, 31 of 65 studies at full-text screening were excluded due to lack of reported IMD incidence. Cohen’s κ at the abstract (0.64) and full text (0.66) screening indicated substantial agreement between reviewers^48^. In total, included RCTs involved 32,572 patients with HER2-positive breast cancer. Four studies^25,52,53,59^ compared dual HER2-targeted therapy to trastuzumab (*n* = 10,103), seven studies^6,55–58,60,65^ compared trastuzumab to chemotherapy or observation (*n* = 13,752), and nine studies^28,29,52–54,61–64^ compared trastuzumab to another HER2-targeted agent (*n* = 13,207). Ten of the eighteen eligible studies involved patients with early stage disease. Median follow-up ranged from 9 months to 11 years from the start of trial therapy. Regular intracranial CT/MRI imaging was reported by only two studies. The characteristics of these trials are presented in Table 1 and Supplementary Table 6.

**Table 1:** Summary of characteristics of included trials.

Trial (Year^a^)	BC stage^b^	Median follow-up (years)	No. HER2 + patients	Role of HER2-targeted agent	Trial arm	Planned duration HER2-targeted therapy	No. patients in arm	No. of CNS events	No. recurrence events^*c*^	OS (HR, 95%CI)	PFS (HR, 95% CI)
NeoALTTO (2012)^1,2^	Early	6.7	455	Neoadjuvant	Lapatinib followed by paclitaxel then concurrent lapatinib and FEC	1 year	154	6	44	0.85 (0.49–1.46)	0.98 (0.64–1.51)
					Trastuzumab followed by paclitaxel then concurrent trastuzumab and FEC	1 year	149	8	45	1 [reference]	1 [reference]
					Lapatinib plus trastuzumab plus paclitaxel followed by lapatinib plus trastuzumab plus FEC	1 year	152	9	38	0.72 (0.41–1.27)	0.81 (0.52–1.26)
ALTTO (2016)^3^	Early	4.5	8381	Adjuvant	Trastuzumab, chemotherapy	1 year	2097	40	301	1 [reference]	1 [reference]
					Lapatinib, chemotherapy	1 year	2100	50	366	1.36 (1.09–1.72)	1.34 (1.13–1.60)
					Trastuzumab then lapatinib, chemotherapy	1 year	2091	48	284	0.91 (0.71–1.16)	0.96 (0.80–1.15)
					Trastuzumab plus lapatinib, chemotherapy	1 year	2093	41	254	0.80 (0.62–1.03)	0.84 (0.70–1.02)
APHINITY (2017)^4^	Early	3.8	4805	Adjuvant	Pertuzumab plus trastuzumab plus taxanes	1 year	2400	45	171	0.89 (0.66–1.21)	0.81 (0.66–1.00)
					Placebo plus trastuzumab plus taxanes	1 year	2404	44	210	1 [reference]	1 [reference]
WJOG6110B/ELTOP (2018)^5^	Advanced	3.7	86	Second or third line	Trastuzumab plus capecitabine	Not specified	43	2	NR	1 [reference]	1 [reference]
					Lapatinib plus capecitabine	Not specified	43	2	NR	0.58 (0.26–1.31)	0.81 (0.55–1.21)
HERA (2005)^6,7^	Early	11	5099	Adjuvant	Trastuzumab (1 year)	1 year	1702	45	505	0.74 (0.64–0.86)	0.76 (0.68–0.86)
					Trastuzumab (2 years)	2 years	1700	32	518	NR	0.77 (0.69–0.87)
					Observation	NA	1697	36	608	1 [reference]	1 [reference]
NSABP B-31/NCCTG N9831 (2005)^8,9^	Early	8.4 (trastuzumab), 8.3(control)	4046	NR	AC then paclitaxel	NA	2018	40	680	1 [reference]	1 [reference]
					AC then paclitaxel plus trastuzumab	1 year	2028	63	473	0.61 (0.52–0.71)	0.58 (0.52–0.66)
BCIRG-006 (2011)^10,11^	Early	10.3	3222	Adjuvant	AC then docetaxel	NA	1073	37	NR	1 [reference]	1 [reference]
					AC then trastuzumab plus docetaxel	1 year	1074	30	NR	0.74 (0.43–1.27)	1.21 (0.74–1.99)
					Docetaxel plus carboplatin plus trastuzumab	1 year	1075	34	NR	NA	NA
FNCLCC-PACS 04 (2009)^12^	Early	3.9	528	Adjuvant	Trastuzumab	1 year	260	11	59	1.27 (0.68–2.38)	0.86 (0.61–1.22)
					Observation	NA	268	8	70	1 [reference]	1 [reference]
CLEOPATRA (2015)^13,14^	Advanced	2.5	808	First line	Pertuzumab plus trastuzumab plus docetaxel	Minimum 18 weeks	402	63	NR	0.68 (0.56–0.84)	0.68 (0.58–0.80)
					Placebo plus trastuzumab plus docetaxel	Minimum 18 weeks	406	62	NR	1 [reference]	1 [reference]
FinHer (2009)^15^	Early	5.2	232	Adjuvant	Docetaxel or vinorelbine then FEC	NA	116	5	31	1 [reference]	1 [reference]
					Docetaxel or vinorelbine concurrent with trastuzumab, then FEC	9 weeks	115	3	27	0.55 (0.27–1.11)	0.65 (0.38–1.12)
CEREBEL (2015)^16^	Advanced	NR	540	Any	Lapatinib plus capecitabine	Not specified	251	8	NR	1 [reference]	1 [reference]
					Trastuzumab plus capecitabine	Not specified	250	12	NR	0.75 (0.61–1.05)	0.77 (0.61–0.96)
NEfERT-T (2016)^17^	Advanced	1.9	479	First line	Neratinib plus paclitaxel	Not specified	242	20	167	1 [reference]	1 [reference]
					Trastuzumab plus paclitaxel	Not specified	237	41	156	0.95 (0.69–1.32)	0.98 (0.79–1.23)
LUX Breast-1 (2016)^18^	Advanced	0.78	508	First or second line	Afatinib plus vinorelbine	Not specified	339	30	180	1 [reference]	1 [reference]
					Trastuzumab plus vinorelbine	Not specified	169	19	74	0.67 (0.51–0.89)	0.91 (.71–1.16)
KATHERINE (2019)^19^	Early	3.5	1486	Adjuvant	Trastuzumab emtansine	42 weeks	743	44	91	1 [reference]	1 [reference]
					Trastuzumab	42 weeks	743	32	165	1.43 (0.95–2.13)	2.00 (1.06–2.56)
GeparQuinto (2012)^20,21^	Early	4.6	620	Neoadjuvant	Trastuzumab plus EC then docetaxel then trastuzumab monotherapy	76 weeks	309	15	60	1 [reference]	1 [reference]
					Lapatinib plus EC then docetaxel then trastuzumab monotherapy	76 weeks	311	13	63	0.76 (0.45–1.28)	1.04 (0.73–1.49)
NCIC CTG MA.31 (2015)^22^	Advanced	1.8	652	First line	Lapatinib plus taxane then lapatinib monotherapy	24 weeks then until progression	326	44	242	1.28 (0.95–1.72)	1.37 (1.13–1.65)
					Trastuzumab plus taxane then trastuzumab monotherapy	24 weeks then until progression	326	52	219	1 [reference]	1 [reference]
Slamon et al. (2001)^23^	Metastatic	2.5	469	First line	(A or E) plus (C or pac)	NA	234	21	NR	1 [reference]	1 [reference]
					(A or E) plus (C or pac) plus trastuzumab	18 weeks or until progression	235	42	NR	0.82 (0.66–1)	0.53 (0.43–0.65)
GBG/BIG 03-05^24^	Metastatic	1.3	156	Second line	Capecitabine	NA	78	5	65	1 [reference]	1 [reference]
					Trastuzumab plus capecitabine	Not specified	78	8	62	0.76 (0.48–1.22)	0.66 (0.43–0.99)

*adv* advanced, *OS* overall survival, *PFS* progression-free survival, *(F)EC* (fluorouracil plus) epirubicin plus cyclophosphamide, *AC* doxorubicin plus cyclophosphamide, *A or E* doxorubicin or epirubicin, *C or pac* cyclophosphamide or paclitaxel.

^a^Year of original trial publication, not the year of separate article reporting on IMD incidence.

^b^Early defined as stage I–II, advanced defined as stage III–IV.

^c^Recurrence events include: locoregional recurrence, distant recurrence, contralateral breast cancer, other second primary cancer, and death without recurrence.

**Fig. 2:**
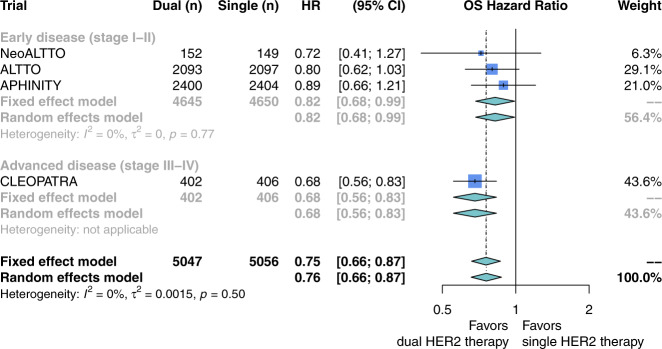
Overall survival with the addition of pertuzumab or lapatinib to trastuzumab for patients with HER2-positive breast cancer. Hazard ratios for overall survival were extracted from eligible studies and pooled using a random-effects model. Studies here are stratified by disease stage: either early (stage I–II) or advanced (stage III–IV). The size of each box represents the weight of each study in the meta-analysis. The vertical solid line represents the point of equivalence between dual and single HER2 therapy. The vertical dashed and dotted lines represent the points of summary for fixed and random effects models, respectively, and the diamonds represent 95% CI for the summary hazard ratios. Analyses were performed with the R programming language^50^ and the R package meta^51^.

**Fig. 3:**
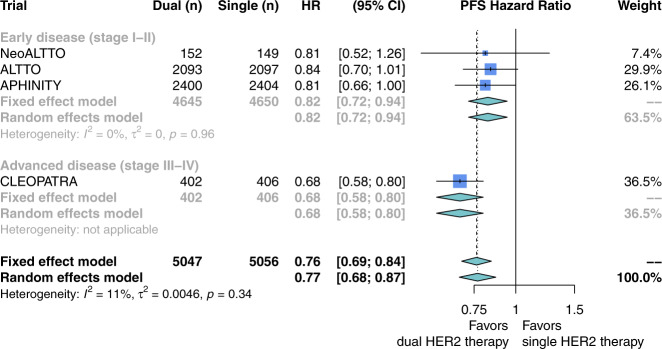
Progression-free survival with the addition of pertuzumab or lapatinib to trastuzumab for patients with HER2-positive breast cancer. Hazard ratios for progression-free survival were extracted from eligible studies and pooled using a random-effects model. Studies here are stratified by disease stage: either early (stage I–II) or advanced (stage III–IV). The size of each box represents the weight of each study in the meta-analysis. The vertical solid line represents the point of equivalence between dual and single HER2 therapy. The vertical dashed and dotted lines represent the points of summary for fixed and random effects models, respectively, and the diamonds represent 95% CI for the summary hazard ratios. Analyses were performed with the R programming language^50^ and the R package meta^51^.

Dual HER2-targeted therapy was associated with prolonged OS (four studies; HR 0.76; 95% CI, 0.66–0.87; Fig. 2; GRADE high) and PFS (four studies; HR 0.77; 95% CI, 0.68–0.87; Fig. 3; GRADE high) compared to single HER2-targeted therapy with trastuzumab. Heterogeneity in these comparisons was low (*I*^2^ = 0% for OS and 11% for PFS). Stratification by disease stage showed dual HER2-targeted therapy was associated with prolonged OS and PFS in both early stage (OS: three studies; HR 0.82; 95% CI, 0.68–0.99; Fig. 2. PFS: three studies; HR 0.82; 95% CI, 0.72–0.94; Fig. 3) and advanced-stage disease (OS: 1 study; HR 0.68; 95% CI, 0.56–0.83; Fig. 2. PFS: 1 study; HR 0.68; 95% CI, 0.58–0.80; Fig. 3).

**Fig. 4:**
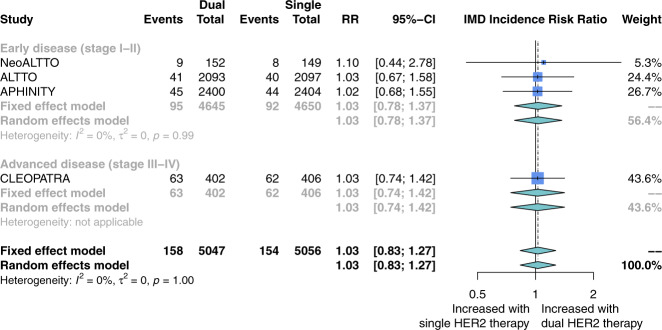
Risk ratio of intracranial metastatic disease with the addition of pertuzumab or lapatinib to trastuzumab for patients with HER2-positive breast cancer. Risk ratios were calculated from the proportion of patients in each study arm who developed the intracranial metastatic disease over the study course. Studies here are stratified by disease stage: either early (stage I–II) or advanced (stage III–IV). The size of each box represents the weight of each study in the meta-analysis. The vertical solid line represents the point of equivalence between dual and single HER2 therapy. The vertical dashed and dotted lines represent the points of summary for fixed and random effects models, respectively, and the diamonds represent 95% CI for the summary relative risks. Analyses were performed with the R programming language^50^ and the R package meta^51^.

The risk of IMD incidence was not different between patients receiving dual versus single HER2-targeted therapy (four studies; RR 1.03; 95% CI, 0.83–1.27; Fig. 4). Heterogeneity in this comparison was low (*I*^2^ = 0%). Subgroup analysis revealed no difference between early stage (three studies; RR 1.03; 95% CI, 0.78–1.37; Fig. 4) and advanced-stage disease (one study; RR 1.03; 95% CI, 0.74–1.42; Fig. 4), or if the dual-therapy included lapatinib as the second agent (two studies; RR 1.04; 95% CI, 0.70–1.54; Supplementary Fig. 1) versus pertuzumab (two studies; RR 1.03; 95% CI, 0.83–1.64; Supplementary Fig. 1).

**Fig. 5:**
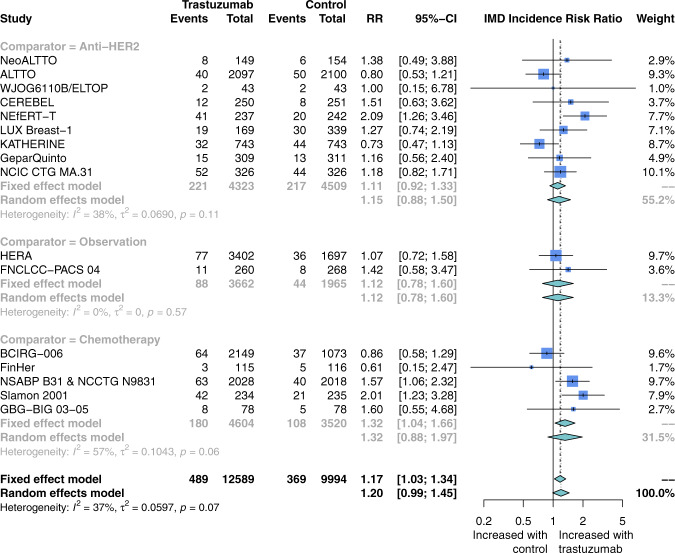
Risk ratio of intracranial metastatic disease in patients receiving trastuzumab versus comparator for HER2-positive breast cancer. Risk ratios were calculated from the proportion of patients in each study arm who developed the intracranial metastatic disease over the study course. Studies here are stratified by comparator regimen: either chemotherapy, observation, or another HER2-targeted agent. The size of each box represents the weight of each study in the meta-analysis. The vertical solid line represents the point of equivalence between trastuzumab and comparators. The vertical dashed and dotted lines represent the points of summary for fixed and random effects models, respectively, and the diamonds represent 95% CI for the summary relative risks. Analyses were performed with the R programming language^50^ and the R package meta^51^.

Patients receiving trastuzumab did not show an increased incidence of IMD compared to another HER2-targeted therapy (nine studies; RR 1.15; 95% CI 0.88–1.50), observation (two studies; RR 1.12; 95% CI 0.78–1.60), or chemotherapy (five studies; RR 1.32; 95% CI, 0.88–1.97) (Fig. 5). Heterogeneity in this comparison was moderate overall (*I*^2^ = 37%), and higher but still moderate in the chemotherapy (*I*^2^ = 57%) and anti-HER2 agent (*I*^2^ = 38%) comparator subgroups. The summary estimate for IMD incidence from pooling the seven studies of trastuzumab monotherapy versus chemotherapy or observation comparators was RR 1.27 (95% CI, 0.95–1.70) (Supplementary Fig. 2). Subgroup analysis of studies of early stage disease showed no difference in IMD incidence (RR 1.01; 95% CI, 0.81–1.26; nine studies; Supplementary Fig. 3) between trastuzumab monotherapy versus chemotherapy, observation, or another HER2-targeted agent. Subgroup analysis of studies of advanced-stage disease showed significantly increased IMD incidence (RR 1.53, 95% CI, 1.19–1.97; seven studies; Supplementary Fig. 4) with trastuzumab monotherapy versus chemotherapy or another HER2-targeted agent.

The overall risk of bias was low in 6/18 (33%) and moderate in 12/18 (66%) included RCTs (Fig. S5). Summary plots for risk of bias showed low to moderate risk for the meta-analyses in this study (Supplementary Fig. 6). Assessment of funnel plots did not indicate publication bias, although these assessments were underpowered in all but one case (Supplementary Figs. 7–11). Sensitivity analysis showed that the findings were robust: comparison of random- and fixed-effects estimates did not render any significant summary estimates insignificant, and iterative omission of each study in the “leave-out-one” analyses did not significantly perturb summary estimates, including one study with follow-up <1 year^29^. GRADE certainty level was “high” for summary estimates (Table 2).

**Table 2:** GRADE summary of findings.

Certainty assessment							Summary of findings				
Participants (studies)	Risk of bias	Inconsistency	Indirectness	Imprecision	Publication bias	Overall certainty of evidence	Study event rates (%)		Relative effect (95% CI)	Anticipated absolute effects	
							With [comparison]	With [intervention]		Risk with [comparison]	Risk difference with [intervention]
Dual-HER2 vs. trastuzumab monotherapy: IMD incidence											
10103 (4 RCTs)	Not serious	Not serious	Not serious	Not serious	None	⨁⨁⨁⨁ HIGH	154/5056 (3.0%)	158/5047 (3.1%)	RR 1.03 (0.83 to 1.27)	30 per 1000	1 more per 1000 (from 5 fewer to 8 more)
Dual-HER2 vs. trastuzumab monotherapy: OS											
10103 (4 RCTs)	Not serious	Not serious	Not serious	Not serious	None	⨁⨁⨁⨁ HIGH	5056 participants	5047 participants	HR 0.76 (0.66 to 0.87) [Dual-HER2 vs. trastuzumab monotherapy: OS]	500 per 1000	90 fewer per 1000 (from 133 fewer to 47 fewer)
Dual-HER2 vs. trastuzumab monotherapy: PFS											
10103 (4 RCTs)	Not serious	Not serious	Not serious	Not serious	None	⨁⨁⨁⨁ HIGH	5056 participants	5047 participants	HR 0.77 (0.68 to 0.87) [Dual-HER2 vs. trastuzumab monotherapy: PFS]	500 per 1000	86 fewer per 1000 (from 124 fewer to 47 fewer)
Trastuzumab monotherapy vs. chemotherapy or observation: IMD incidence											
13751 (8 RCTs)	Not serious	Not serious	Not serious	Not serious	None	⨁⨁⨁⨁ HIGH	152/5485 (2.8%)	268/8266 (3.2%)	RR 1.27 (0.95 to 1.70)	28 per 1000	7 more per 1000 (from 1 fewer to 19 more)

*CI* Confidence interval, *RR* Riskratio, *HR* Hazard Ratio.

## Discussion

Our study found prolonged OS and PFS without a significant difference in IMD incidence, with the addition of lapatinib or pertuzumab to trastuzumab for patients with HER2-positive breast cancer. Although there is mixed evidence for the ability of lapatinib to penetrate the blood-brain barrier^66–68^, our pooled analysis of two studies failed to show a difference in IMD incidence between lapatinib plus trastuzumab versus trastuzumab alone. While our study was not designed to assess this comparison, future reporting may clarify a role for HER2-targeted TKIs in IMD prevention. Of note, several high-profile trials (HER2CLIMB^30^, DESTINY^34^, TH3RESA^69^, EMILIA^24^, MARIANNE^70^) were captured in the literature search but did not meet inclusion criteria because they either have not yet reported IMD incidence, or featured absent or ineligible comparators.

Our study did not find a significant difference in IMD incidence between patients receiving dual anti-HER2 therapy versus trastuzumab alone. We also did not find a difference comparing trastuzumab with chemotherapy or observation, but this is in contradistinction to previous meta-analyses of RCTs^17–21^. One possible explanation for this difference is that our meta-analysis for this outcome involved more patients and longer follow-up for events to accrue. Further, previous meta-analyses included only studies of early stage disease, but our subgroup analysis of this population did not show a significant difference in IMD incidence between trastuzumab monotherapy and chemotherapy or observation (HR 1.01; 95% CI, 0.81–1.26; *p* = 0.92; Supplementary Fig. 3). Conversely, our subgroup analysis of advanced-stage disease showed an association between trastuzumab monotherapy and increased incidence of IMD, compared to chemotherapy or another HER2-targeted agent (HR 1.53; 95% CI, 1.19–1.97; *p* = 0.001; Supplementary Fig. 4).

These subgroup findings suggest that IMD may be more likely among patients with advanced-stage HER2+ breast cancer who receive trastuzumab monotherapy as compared to chemotherapy or another HER2-targeted agent. These findings also suggest that the impact of dual- versus single-agent HER2-therapy on IMD incidence could be different for patients with advanced- versus early stage disease. Our study did not detect such a difference (*p* = 0.97; Fig. 4), but this could be due to the small number of studies (advanced: *n* = 1, early: *n* = 3) in this comparison. Our subgroup analysis of early stage disease did not show a difference in IMD incidence between patients receiving trastuzumab monotherapy versus chemotherapy or observation. However, this does not rule out the possibility that trastuzumab monotherapy influences IMD incidence for these patients, as the rarity of IMD in these studies renders this estimate susceptible to confounding.

GRADE certainty level was high for summary estimates, although some indirectness may be present in the estimates due to pooling studies of early- and advanced-stage disease. As well, the magnitudes of summary effect sizes were modest.

Our study has several limitations. First, our study did not distinguish between the incidence of IMD overall versus the incidence of IMD as the first site of recurrence. This may have biased our results towards a lower estimate of overall IMD incidence, although one would expect this bias to impact both experimental and control arms similarly. The impact of HER2-targeted agents on IMD incidence overall versus as the first site of recurrence remains underexplored^71^. Second, patients were pooled from treatment arms that received the same chemotherapy plus trastuzumab combination, but in concurrent or sequential order, or for different durations. This was necessary because there were few studies available for pooling. However, omitting these studies one at a time in the sensitivity analysis did not change our conclusions. Third, our study did not take into account the duration of HER2-targeted regimens, which is a potential confounder, although data from the HERA study suggest no difference in OS or IMD incidence between 1-year and 2-year trastuzumab treatment arms^55^. Fourth, IMD was not the main outcome of most RCTs, and RR was reported instead of HR. For patients with non-metastatic breast cancer, the occurrence of IMD may take years to develop, and differences in time-to-event comparisons may be obscured by comparing RR across studies with different follow-up durations. Future studies are needed to establish whether HER2-targeted therapy delays rather than prevent IMD. Fifth, blinding was infrequent in the included studies, which could have resulted in an overestimation of survival benefit for patients receiving the study regimen in included studies due to performance bias. Sixth, regular intracranial imaging was reported by only two studies, which could have resulted in an underestimation of a difference in IMD incidence between groups. Seventh, this study was unable to address the impact of the therapy lines on outcomes due to too few studies. Finally, our results may have been impacted by the biases associated with RCTs. Attrition bias may have led to underestimates of IMD incidence following HER2-targeted therapy, although this is mitigated by the majority of included studies reporting analyses based on intention-to-treat populations^25,28,52,54,55,57–59,62,72–76^. Although most studies excluded at full-text review did not report IMD incidence despite evaluating the incidence of other safety outcomes in patients receiving HER2-targeted agents, we were unable to demonstrate publication bias with the small number of studies included.

While other meta-analyses have assessed survival and safety between dual- and single-agent HER2-targeted regimens^77–79^, our study compares IMD incidence in these groups and provides the most recent update on the risk of IMD incidence following trastuzumab monotherapy. Our data suggest prolonged survival may not be associated with increased risk of IMD incidence when comparing dual to single HER2-targeted therapy, and when comparing trastuzumab to chemotherapy and observation in early stage disease. In an effort to maximize the knowledge gained from randomized treatment allocation, follow-up studies of these trials focusing on the incidence of IMD should be conducted. In addition, the incidence of IMD should be prospectively collected in future studies that involve patients who are at high risk of central nervous system metastases.

## Conclusions

Dual-agent HER2-targeted therapy for eligible patients with breast cancer is associated with prolonged survival without increased risk of IMD compared to trastuzumab. Together, these findings suggest dual HER2-targeted therapy is associated with decreased IMD risk per unit time. The incidence of IMD among patients receiving trastuzumab was not greater than those who received other HER2-targeted agents, chemotherapy, or observation in the setting of early stage disease. Future trials should monitor and report IMD incidence to assess whether the novel and existing systemic therapies may further impact IMD epidemiology across breast cancer subtypes.

## Supplementary information

Supplementary Materials

## Data Availability

The authors declare that the data supporting the findings of this study are available within the paper and its supplementary information files.

## Code availability

The code developed during this study is available upon reasonable request. Analyses were performed using the R programming language version 3.6.1 (R Foundation for Statistical Computing, Vienna, Austria).
