# Supplementary material for: Dual- versus single-agent HER2 inhibition and incidence of intracranial metastatic disease: a systematic review and meta-analysis
Source: NPJ Breast Cancer. 2021 Feb 18;7:17. doi: 10.1038/s41523-021-00220-0 (PMC7892568; doi:10.1038/s41523-021-00220-0)
Supplement: Supplementary file 1 — Supplementary Materials [file 41523_2021_220_MOESM1_ESM.pdf]

Supplementary Materials

Dual-HER2 inhibition with trastuzumab and incidence of intracranial metastatic disease: a systematic review and meta-analysis.

Anders Wilder Erickson, BSc, Steven Habbous, PhD, Christianne Hoey, MSc, Katarzyna Jerzak, MD, Sunit Das, MD, PhD

Supplementary Methods

A formal protocol for this study was not written or registered.

Data extraction and quality assessment

Investigators from the four included RCTs assessing dual versus single HER2 therapy were contacted for individual participant data, which was not available at the time of contact. Risk of bias was assessed at the study level using the Cochrane Risk of Bias 2 tool.[1]

Data synthesis and analysis

Study estimates were pooled with the inverse variance method using random-effects models on the assumption that the included studies did not stem from the same population, i.e. that there was variation between studies based on interventions, patient populations, and methods.[2] A restricted maximum-likelihood (REML) estimator was used for τ^2. Overall survival (OS) and progression-free survival (PFS) hazard ratios were selected from study arms that compared concurrent dual HER2-targeted therapy to trastuzumab, not sequential dual therapy. Patients receiving the same therapy in different sequences within a study were pooled for analysis into one treatment arm. This occurred in the analyses of the HERA and BCIRG-006 trials.

Ethics review

This study was not reviewed by any ethics review board as it did not contain live subjects, nor samples, nor individual-level patient data.

Supplementary Tables

Supplementary Table 1. MEDLINE query – PubMed interface

Years 1966 to 2020 March 25

#	Searches
1	Trastuzumab[MeSH Terms]
2	Trastuzumab[All Fields]
3	Herceptin[All Fields]
4	1 or 2 or 3
5	Breast[MeSH Terms]
6	Breast[All Fields]
7	5 or 6
8	Adenocarcinoma[MeSH Terms]
9	Adenocarcinoma[All Fields]
10	Neoplasms[MeSH Terms]
11	Neoplasms[All Fields]
12	Cancer[All Fields]
13	8 or 9 or 10 or 11 or 12
14	4 and 7 and 13, filter Randomized Controlled Trials, English language

Supplementary Table 2. EMBASE query – Ovid interface
Database(s): **Embase Classic+Embase** 1947 to 2020 March 24
Search Strategy:

#	Searches	Results
1	Clinical Trial/	991146
2	Randomized Controlled Trial/	598948
3	multicenter study/	246677
4	Phase 3 clinical trial/	46300
5	Phase 4 clinical trial/	3815
6	exp RANDOMIZATION/	86861
7	Single Blind Procedure/	38384
8	Double Blind Procedure/	173377
9	Crossover Procedure/	62941
10	PLACEBO/	358652
11	randomi?ed controlled trial$.tw.	224036
12	rct.tw.	36311
13	(random$ adj2 allocat$).tw.	42592
14	single blind$.tw.	24701
15	double blind$.tw.	212890
16	((treble or triple) adj blind$).tw.	1170
17	placebo$.tw.	310286
18	Prospective Study/	591741
19	controlled clinical trial/	464061
20	or/1-19	2320191
21	Case Study/	77040
22	case report.tw.	433926
23	abstract report/ or letter/	1136346
24	Conference proceeding.pt.	0
25	Conference abstract.pt.	3732365
26	Editorial.pt.	646604
27	Letter.pt.	1106501
28	Note.pt.	790068
29	or/21-28	6752726
30	20 not 29	1734034
31	trastuzumab.ti.	7616
32	herceptin.ti.	688
33	31 or 32	8074
34	30 and 33	1215
35	limit 34 to (human and english language and randomized controlled trial and article)	305
36	supplement.so.	1216747
37	35 not 36	286

Supplementary Table 3. CENTRAL query – Wiley interface

Last Saved: 25/03/2020 13:19:58

ID	Search	Results
#1	(trastuzumab):ti	1528
#2	(herceptin):ti	192
#3	#1 or #2	1626
#4	breast	46457
#5	cancer	171321
#6	adenocarcinoma	10295
#7	#5 or #6	172395
#8	MeSH descriptor: [Central Nervous System Neoplasms] explode all trees and with qualifier(s): [secondary - SC]	352
#9	MeSH descriptor: [Central Nervous System Neoplasms] this term only	178
#10	MeSH descriptor: [Cerebral Cortex] this term only	1033
#11	MeSH descriptor: [Brain] this term only	4655
#12	#9 or #10 or #11	5780
#13	MeSH descriptor: [Neoplasm Metastasis] this term only	3061
#14	#12 and #13	9
#15	((brain* or intra*cranial or cerebral or cerebrum or crani* or skull or cns or leptomening* or mening* or posterior fossa or frontal lobe or parietal lobe or temporal lobe or occipital lobe or insula* or cortex or cortic* or encephal* or hippocamp* or gyrus or limbic or dentate or white matter or gr*y matter) NEAR/3 (metasta* or (secondar* NEAR/3 (malig* or cancer or disease or neoplas* or tumo*r* or carcinoma* or spread*)))):ti,ab,kw	2285
#16	#8 or #14 or #15	2332
#17	#3 and #5 and #7 and #16 in Trials	79

Supplementary Table 4. Inclusion and exclusion criteria for abstract/title screening

**Design**	
**Include:**<br>• Randomized-controlled trials	**Exclude:**<br>• Non-randomized controlled trials<br>• Cohort studies<br>• Case-control studies<br>• Case series<br>• Case reports<br>• Systematic review / meta-analysis
**Population**	
**Include:**<br>• HER2+ breast cancer<br>• Studies containing only a proportion of patients with HER2+ breast cancer, but for whom outcomes are specifically reported	**Exclude:**<br>• Studies without HER2+ breast cancer patients<br>• Studies combining analysis of patients with varying breast cancer subtypes, in which no reported outcomes are specific to patients with HER2+ disease
**Intervention**	
**Include:**<br>• Trastuzumab monotherapy<br>• Trastuzumab in combination with another HER2-targeted agent (i.e. "dual" HER2-therapy)	**Exclude:**<br>• Biosimilar trastuzumab
**Comparators**	
**Include:**<br>• If intervention = trastuzumab monotherapy:<br>o Chemotherapy<br>o HER2-targeted medical therapy (non-trastuzumab)<br>o Placebo<br>o Observation<br>• If intervention = dual HER2 therapy: Trastuzumab monotherapy	**Exclude:**<br>• Studies reporting on biosimilar trastuzumab<br>• Studies only comparing different dosages or sequences of trastuzumab<br>• Studies only comparing different dosages or sequences of the combination of trastuzumab plus another HER2-targeted agent<br>• Studies comparing trastuzumab to surgical intervention<br>• Studies comparing trastuzumab to radiotherapeutic intervention

Supplementary Table 5. Inclusion and exclusion criteria for full text review

**Outcomes**	
**Include:**<br>• Reports incidence of intracranial metastatic disease following initiation of trastuzumab	**Exclude:**<br>• Does not report incidence of intracranial metastatic disease<br>• Only reports baseline prevalence of intracranial metastatic disease<br>• Reports results for same patients as in a separate publication that has already been included or excluded<br>• Full text unavailable<br>• Trial not yet complete

Supplementary Table 6. Additional details from included trials

Trial	Treatment arm	Prior treatment	Additional notes	CNS MRI/CT freq.
NeoALTTO [3,4]	L: Lapatinib 1500mg/day x 6 wks, followed by weekly same dose lapatinib plus paclitaxel (80mg/m^2) x 12 weeks before definitive breast surgery. Then 3 cycles FEC every 3 weeks followed by same dose lapatinib until 52 weeks.	NR	NeoALTTO: "According to a protocol amendment in 2008, the lapatinib dose was reduced to 750 mg/day in combination with paclitaxel and trastuzumab because of toxicity (diarrhoea). In total, 54 of 152 patients received this reduced dose."	NR
	H: Trastuzumab loading dose 4mg/kg, then 2mg/kg/wk x 6 weeks, followed by weekly same dose trastuzumab plus paclitaxel (80mg/m^2) x 12 weeks before definitive breast surgery. Then 3 cycles FEC every 3 weeks followed by same dose trastuzumab until 52 weeks.			
	L+H: Lapatinib 1000mg/day plus trastuzumab 1000mg/day x 6 weeks, followed by weekly same doses lapatinib and trastuzumab plus paclitaxel (80mg/m^2) x 12 weeks before definitive breast surgery. Then 3 cycles FEC every 3 weeks followed by same dose lapatinib and trastuzumab until 52 weeks.			
ALTTO [5]	H: Intravenous trastuzumab at a loading dose of 4 mg/kg once and then 2 mg/kg weekly during chemotherapy, or at a loading dose of 8 mg/kg once and then 6 mg/kg every 3 weeks when given alone until 52 weeks.	NR	ALTTO: HER2-targeted therapies were administered to each patient in relationship to chemotherapy among one of three possible designs: 1) following completion of all 12–18 weeks' chemotherapy, 2) following completion of 9–12 week's anthracycline chemotherapy, and concurrent to 12 weeks' taxane chemotherapy, or 2B) concurrent to 18 weeks' docetaxel plus carboplatin.	NR
	L: Oral lapatinib 750 mg/day during chemotherapy and 1,500 mg/day when given alone until 52 weeks.			
	H–>L: 12 weekly doses of intravenous trastuzumab as in (H) arm, followed after a 6-week washout by 34 weeks of oral lapatinib at 1,500 mg/day until 52 weeks.			
	L+H: Lapatinib plus trastuzumab, with trastuzumab as in (H) arm and lapatinib at 750 mg/day during chemotherapy with an escalation to 1,000 mg/day at chemotherapy completion until 52 weeks.			

APHINITY[6]	P + H: Pertuzumab 840mg loading dose IV then 420mg IV every 3 weeks and trastuzumab 8mg/kg IV every 3 weeks concurrent with taxane chemotherapy for maximum 18 cycles until 52 weeks.	NR	APHINITY: "Anti-HER2 treatment was given in combination with chemotherapy according to one of the following schedules: 3 or 4 cycles (every 3 weeks) of 5-fluorouracil plus either epirubicin or doxorubicin plus cyclophosphamide, followed by 3 or 4 cycles (every 3 weeks) of docetaxel or 12 weekly cycles of paclitaxel; 4 cycles (every 3 weeks or every 2 weeks) of cyclophosphamide plus either doxorubicin or epirubicin, followed by either 4 cycles (every 3 weeks) of docetaxel or 12 weekly cycles of paclitaxel; or 6 cycles (every 3 weeks) of docetaxel plus carboplatin."	NR
	Plac + H: Placebo and trastuzumab 8mg/kg IV every 3 weeks concurrent with taxane chemotherapy for maximum 18 cycles until 52 weeks.			
WJOG6110 B/ELTOP[7]	H+C: Trastuzumab 4 mg/kg loading dose then 2 mg/kg weekly, or 8 mg/kg loading dose then 6 mg/kg every 3 weeks, and capecitabine 2500 mg/m^2/day on days 1–14 every 3 weeks.	Trastuzumab and taxanes		"Brain MRI or CT was performed at baseline and every 6 weeks in patients with brain metastases and every 12 weeks in patients without brain metastases."
	L+C: Lapatinib 1250mg/day and capecitabine 2000 mg/m^2/day on days 1–14 every 3 weeks.			
HERA[8,9]	H1: Trastuzumab 8mg/kg loading dose IV then 6mg/kg every 3 weeks until 52 weeks.	Chemotherapy, definitive surgery, and radiotherapy, if applicable		NR
	H2: Trastuzumab 8mg/kg loading dose IV then 6mg/kg every 3 weeks until 2 years.			
	Obs: Observation.			

NSABP B-31 / NCCTG N9831[10,11]	AC->pac: doxorubicin 60mg/m^2 IV and cyclophosphamide 600mg/m^2 IV every 3 weeks for 4 cycles then either paclitaxel 175mg/m^2 IV every 3 weeks or paclitaxel 80mg/m^2 IV every week for 4 cycles.	NR	NSABP B-31 / NCCTG N9831: Extracted results are from the final planned joint analysis (Perez, 2014) of arms A and C of the N9831trial, and arms 1 and 2 of the B-31 trial.	NR
	AC->H+pac: doxorubicin 60mg/m^2 IV and cyclophosphamide 600mg/m^2 IV every 3 weeks for 4 cycles, then either paclitaxel 175mg/m^2 IV every 3 weeks or paclitaxel 80mg/m^2 IV every week for 12 weeks, concurrent with trastuzumab 4mg/kg IV loading dose then 2mg/kg weekly for 12 weeks, then same dose trastuzumab until 1 year total trastuzumab.			
BCIRG-006[12,13]	AC->doc: Doxorubicin 60 mg/m2 and cyclophosphamide 600 mg/m^2 every 3 weeks for 4 cycles, followed by docetaxel 100 mg/m^2 every 3 weeks for 4 cycles.	NR	BCIRG-006: Median follow up, recurrence values, and hazard ratios for OS and DFs extracted from Chan 2018. Hazard ratios compare pooled trastuzumab arms versus chemotherapy arm.	NR
	AC->doc+H: Doxorubicin 60 mg/m2 and cyclophosphamide 600 mg/m^2 every 3 weeks for 4 cycles, followed by docetaxel 100 mg/m^2 every 3 weeks for 4 cycles and trastuzumab loading dose 4mg/kg then 2mg/kg weekly during for 12 weeks then 6mg/kg every 3 weeks until 52 weeks' trastuzumab treatment.			
	Doc+carb+H: docetaxel 75 mg/m^2 plus carboplatin (area under the curve 6mg/mL/min) every 3 weeks for 6 cycles concurrent with trastuzumab loading dose 4mg/kg then 2mg/kg weekly during for 18 weeks then 6mg/kg every 3 weeks until 52 weeks' trastuzumab treatment.			
FNCLCC-PACS 04[14]	H: Trastuzumab loading dose 8mg/kg then 6mg/kg every 3 weeks until 52 weeks.	Chemotherapy, radiotherapy		NR
	Obs: Observation.			
CLEOPATRA[15,16]	P+H+doc: Pertuzumab loading dose 840mg on day 1 of cycle 1 then 420mg on day 1 of subsequent cycles, with trastuzumab loading dose 8mg/kg on day 2 of cycle 1, then	No chemotherapy or HER2-		"CT or MRI scans of the

	6mg/kg on day 1 of subsequent cycles, and with docetaxel 75mg/m^2 on day 2 of cycle 1 and on day 1 of subsequent cycles. At least 6 cycles recommended.	targeted therapy for metastatic disease	CLEOPATRA: Median follow up, recurrence values extracted from Swain 2014 Ann Onc. Hazard ratios from Swain 2015 NEJM.	brain and/or spine were carried out when CNS metastases were clinically suspected only.”
	Plac+H+doc: Placebo on day 1 of cycle 1 then on day 1 of subsequent cycles, with trastuzumab loading dose 8mg/kg on day 2 of cycle 1, then 6mg/kg on day 1 of subsequent cycles, and with docetaxel 75mg/m^2 on day 2 of cycle 1 and on day 1 of subsequent cycles. At least 6 cycles recommended.			
FinHer[17]	Ch: Either docetaxel 100mg/m^2 every 3 weeks or, vinorelbine 25mg/m^2 on days 1, 8, 15 every 3 weeks, for 3 cycles. Then fluorouracil 600mg/m^2, epirubicin 60mg/m^2, and cyclophosphamide 600mg/m^2 every 3 weeks for 3 cycles.	NR		NR
	H+Ch: Either docetaxel 100mg/m^2 every 3 weeks or, vinorelbine 25mg/m^2 on days 1, 8, 15 every 3 weeks, for 3 cycles. Concurrently, trastuzumab loading dose 4mg/kg then 2mg/kg weekly for 9 weeks. Then fluorouracil 600mg/m^2, epirubicin 60mg/m^2, and cyclophosphamide 600mg/m^2 every 3 weeks for 3 cycles.			
CEREBEL[18]	L+C: Lapatinib 1250mg/day and capecitabine 2000 mg/m^2/day on days 1–14 every 3 weeks.	Some patients previously received chemotherapy and trastuzumab-containing regimens.	CEREBEL: Hazard ratios were back-calculated to facilitate comparison of the trastuzumab regimen to the lapatinib regimen from the following results presented in Pivot et al. 2015 JCO. HR for OS, 1.34 (95% CI, 0.95–1.64); HR for PFS, 1.30 (95% CI, 1.04–1.64).	“Efficacy assessments, including brain magnetic resonance imaging (MRI) scans, were performed at screening, every 12 weeks until week 84, and
	H+C: Trastuzumab 8 mg/kg loading dose then 6 mg/kg every 3 weeks, and capecitabine 2500 mg/m^2/day on days 1–14 every 3 weeks.			

				every 24 weeks thereafter. CNS symptoms were assessed every 3 weeks.”
NEfERT-T[19]	N+pac: Neratinib 240mg oral daily plus paclitaxel 80mg/m^2 iV on days 1, 8, and 15 every 28 days.	None	NEfERT-T: Hazard ratios were back-calculated to facilitate comparison of the trastuzumab regimen to the neratinib regimen from the following results presented in Awada et al. 2016. JAMA Onc. HR for OS, 1.05 (95% CI, 0.76–1.45); HR for PFS, 1.02 (95% CI, 0.81–1.27).	“…Additional imaging (ie, bone scans, contrast-enhanced CT or MRI of the brain or other sites) was per-formed at baseline and repeated every 8 weeks if disease was present and/or if clinically indicated.”
	T+pac: Trastuzumab 4mg/kg loading dose IV then 2mg/kg on days 1, 8, 15, and 22 every 28 days plus paclitaxel 80mg/m^2 IV on days 1, 8, and 15 every 28 days.			
LUX Breast-1[20]	Af+vin: Afatinib 40mg oral daily plus vinorelbine 25mg/m^2 IV weekly.	Previous adjuvant or first line trastuzumab	LUX-Breast 1: Hazard ratios were back-calculated to facilitate comparison of the trastuzumab regimen to the afatinib regimen from the following results presented in Harbeck et al. 2016 Lancet Onc. HR for OS, 1.48 (95% CI, 1.12–1.95); HR for PFS, 1.10 (95% CI, 0.86–1.41).	NR
	H+vin: Trastuzumab loading dose 4mg/kg IV then 2mg/kg IV weekly plus vinorelbine 25mg/m^2 IV weekly.			
KATHERINE[21]	T-DM1: Trastuzumab emtansine 3.6mg/kg IV every 3 weeks for 14 cycles.	Previous surgery and neoadjuvant chemotherapy	KATHERINE: Hazard ratios were back-calculated to facilitate comparison of the trastuzumab regimen to the trastuzumab-emtansine regimen from the following	NR

		plus HER2-targeted therapy	results presented in von Minckwitz et al. 2019 NEJM. HR for OS, 0.70 (95% CI, 0.47–1.05); HR for DFS, 0.50 (95% CI, 0.39–0.94).	
	H: Trastuzumab 8mg/kg loading dose then 6mg/kg IV every 3 weeks for 14 cycles.			
GeparQuinto[22,23]	ECH->TH: Trastuzumab 8mg/kg IV loading dose then 6mg/kg every 3 weeks for 8 cycles, concurrent with epirubicin 90mg/m^2 and cyclophosphamide 600mg/m^2 every 3 weeks for 4 cycles, then docetaxel 100mg/m^2 every 3 weeks for 4 cycles. Then surgery, then 1 year of trastuzumab.	None		NR
	ECL->TL: Lapatinib 1000–1250mg daily 8 cycles of 3 weeks, concurrent with epirubicin 90mg/m^2 and cyclophosphamide 600mg/m^2 every 3 weeks for 4 cycles, then docetaxel 100mg/m^2 every 3 weeks for 4 cycles. Then surgery, then 1 year of trastuzumab.			
NCIC CTG MA.31[24]	L+tax->L: Lapatinib 1250mg daily concurrent with either paclitaxel IV 80mg/m^2 on days 1, 8, 15 of a 28-day cycle, or docetaxel 75mg/m^2 every 3 weeks for 24 weeks. Then lapatinib 1500mg monotherapy.	Some patients previously received chemotherapy, endocrine therapy, or anti-HER2 therapy, but not in the metastatic setting.	NCIC CTG MA.31: All outcomes extracted for the ITT population (n = 652), not the centrally confirmed HER2+ population (n = 537).	NR
	H+tax->H: Either trastuzumab IV 4mg/kg loading dose then 2mg/kg weekly with paclitaxel IV 80mg/m^2 on days 1, 8, 15 of a 28-day cycle for 24 weeks, or trastuzumab IV 8mg/kg loading dose then 6mg/kg every 3 weeks with docetaxel 75mg/m^2 every 3 weeks for 24 weeks. Then trastuzumab monotherapy 6mg/kg every 3 weeks.			

GBG/BIG 03-05	Capecitabine: capecitabine 1250mg/m^2 q12h on days 1–14 every 3 weeks until progression.	Previous trastuzumab	GBG/BIG 03-05: HR for OS is unadjusted, HR for PFS is adjusted.	NR
	H+capecitabine: capecitabine 1250mg/m^2 q12h on days 1–14 every 3 weeks plus trastuzumab 6mg/kg every 3 weeks until progression.			
Slamon et al. 2001[25]	Ch: doxorubicin 60mg/m^2 or epirubicin 75mg/m^2, plus cyclophosphamide 600mg/m^2 or paclitaxel 175mg/m^2 every 3 weeks for 6 cycles, plus additional cycles at investigator's discretion.	No chemotherapy or anti-HER2 therapy in the metastatic setting.		NR
	H+Ch: doxorubicin 60mg/m^2 or epirubicin 75mg/m^2, plus cyclophosphamide 600mg/m^2 or paclitaxel 175mg/m^2 every 3 weeks for 6 cycles, plus trastuzumab 4mg/kg loading dose then 2mg/kg weekly until disease progression.			

Supplementary Figures

Study	Dual Events	Dual Total	Single Events	Single Total	RR	95%–CI	Weight
**Lapatinib**							
NeoALTTO	9	152	8	149	1.10	[0.44; 2.78]	5.3%
ALTTO	41	2093	40	2097	1.03	[0.67; 1.58]	24.4%
**Fixed effect model**	**50**	**2245**	**48**	**2246**	**1.04**	**[0.70; 1.54]**	**--**
**Random effects model**					**1.04**	**[0.70; 1.54]**	**29.7%**
Heterogeneity: $I^2 = 0\%$, $\tau^2 = 0$, $p = 0.89$							
**Pertuzumab**							
APHINITY	45	2400	44	2404	1.02	[0.68; 1.55]	26.7%
CLEOPATRA	63	402	62	406	1.03	[0.74; 1.42]	43.6%
**Fixed effect model**	**108**	**2802**	**106**	**2810**	**1.03**	**[0.80; 1.32]**	**--**
**Random effects model**					**1.03**	**[0.80; 1.32]**	**70.3%**
Heterogeneity: $I^2 = 0\%$, $\tau^2 = 0$, $p = 0.99$							
**Fixed effect model**	**158**	**5047**	**154**	**5056**	**1.03**	**[0.83; 1.27]**	**--**
**Random effects model**					**1.03**	**[0.83; 1.27]**	**100.0%**
Heterogeneity: $I^2 = 0\%$, $\tau^2 = 0$, $p = 1.00$							

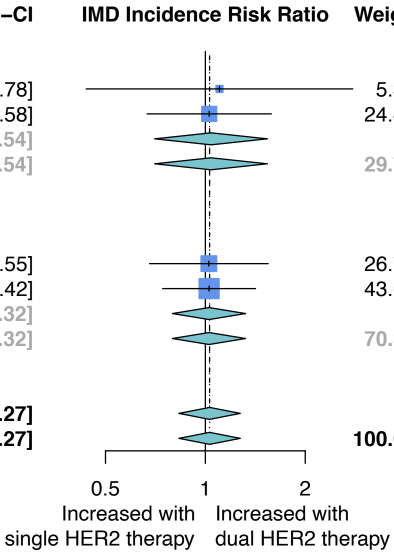

Supplementary Figure 1. Risk ratio of intracranial metastatic disease with the addition of pertuzumab or lapatinib to trastuzumab for patients with HER2-positive breast cancer. Risk ratios were calculated from the proportion of patients in each study arm who developed intracranial metastatic disease over the study course. Studies here are stratified by second HER2-targeted agent: either lapatinib or pertuzumab. The size of each box represents the weight of the contribution of each study to the weight of sample in the meta-analysis. The vertical dashed lines represent the points of summary for fixed and random effects models, and the diamonds represent 95% CI for the summary relative risks. Analyses were performed with the R programming language[26] and the R package meta.[27]

Study	Trastuzumab Events	Trastuzumab Total	Control Events	Control Total	RR	95%−CI	Weight
Comparator = Observation							
HERA	77	3402	36	1697	1.07	[0.72; 1.58]	21.6%
FNCLCC−PACS 04	11	260	8	268	1.42	[0.58; 3.47]	8.1%
Fixed effect model	88	3662	44	1965	1.12	[0.78; 1.60]	−−
Random effects model					1.12	[0.78; 1.60]	29.7%
Heterogeneity: $I^2 = 0\%$, $\tau^2 = 0$, $p = 0.57$							
Comparator = Chemotherapy							
BCIRG−006	64	2149	37	1073	0.86	[0.58; 1.29]	21.3%
FinHer	3	115	5	116	0.61	[0.15; 2.47]	3.8%
NSABP B31 & NCCTG N9831	63	2028	40	2018	1.57	[1.06; 2.32]	21.6%
Slamon 2001	42	234	21	235	2.01	[1.23; 3.28]	17.6%
GBG−BIG 03−05	8	78	5	78	1.60	[0.55; 4.68]	6.0%
Fixed effect model	180	4604	108	3520	1.32	[1.04; 1.66]	−−
Random effects model					1.32	[0.88; 1.97]	70.3%
Heterogeneity: $I^2 = 57\%$, $\tau^2 = 0.1043$, $p = 0.06$							
**Fixed effect model**	**268**	**8266**	**152**	**5485**	**1.25**	**[1.03; 1.52]**	**−−**
**Random effects model**					**1.27**	**[0.95; 1.70]**	**100.0%**
Heterogeneity: $I^2 = 41\%$, $\tau^2 = 0.0607$, $p = 0.12$							

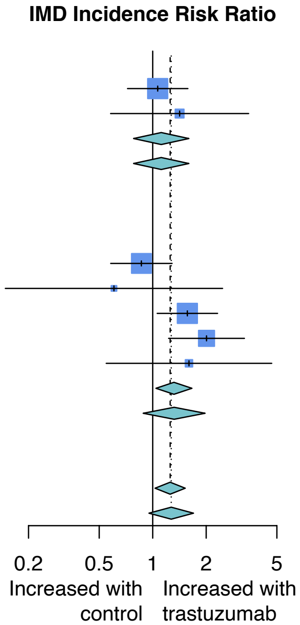

Supplementary Figure 2. Risk ratio of intracranial metastatic disease in patients receiving trastuzumab versus chemotherapy or observation for HER2-positive breast cancer. Risk ratios were calculated from the proportion of patients in each study arm who developed intracranial metastatic disease over the study course. Studies here are stratified by comparator regimen: either chemotherapy or observation. The size of each box represents the weight of the contribution of each study to the weight of sample in the meta-analysis. The vertical dashed lines represent the points of summary for fixed and random effects models, and the diamonds represent 95% CI for the summary relative risks. Analyses were performed with the R programming language[26] and the R package meta.[27]

Study	Trastuzumab Events	Trastuzumab Total	Control Events	Control Total	RR	95%-CI	Weight
**Comparator = Anti–HER2**							
NeoALTTO	8	149	6	154	1.38	[0.49; 3.88]	4.0%
ALTTO	40	2097	50	2100	0.80	[0.53; 1.21]	16.0%
KATHERINE	32	743	44	743	0.73	[0.47; 1.13]	14.7%
GeparQuinto	15	309	13	311	1.16	[0.56; 2.40]	7.3%
**Fixed effect model**	**95**	**3298**	**113**	**3308**	**0.84**	**[0.64; 1.10]**	**--**
**Random effects model**					**0.84**	**[0.64; 1.10]**	**42.0%**
Heterogeneity: $I^2 = 0\%$, $\tau^2 = 0$, $p = 0.55$							
**Comparator = Observation**							
HERA	77	3402	36	1697	1.07	[0.72; 1.58]	16.9%
FNCLCC–PACS 04	11	260	8	268	1.42	[0.58; 3.47]	5.2%
**Fixed effect model**	**88**	**3662**	**44**	**1965**	**1.12**	**[0.78; 1.60]**	**--**
**Random effects model**					**1.12**	**[0.78; 1.60]**	**22.1%**
Heterogeneity: $I^2 = 0\%$, $\tau^2 = 0$, $p = 0.57$							
**Comparator = Chemotherapy**							
BCIRG–006	64	2149	37	1073	0.86	[0.58; 1.29]	16.6%
FinHer	3	115	5	116	0.61	[0.15; 2.47]	2.3%
NSABP B31 & NCCTG N9831	63	2028	40	2018	1.57	[1.06; 2.32]	16.9%
**Fixed effect model**	**130**	**4292**	**82**	**3207**	**1.14**	**[0.87; 1.50]**	**--**
**Random effects model**					**1.08**	**[0.65; 1.82]**	**35.9%**
Heterogeneity: $I^2 = 61\%$, $\tau^2 = 0.1166$, $p = 0.07$							
**Fixed effect model**	**313**	**11252**	**239**	**8480**	**1.01**	**[0.85; 1.19]**	**--**
**Random effects model**					**1.01**	**[0.81; 1.26]**	**100.0%**
Heterogeneity: $I^2 = 23\%$, $\tau^2 = 0.0339$, $p = 0.24$							

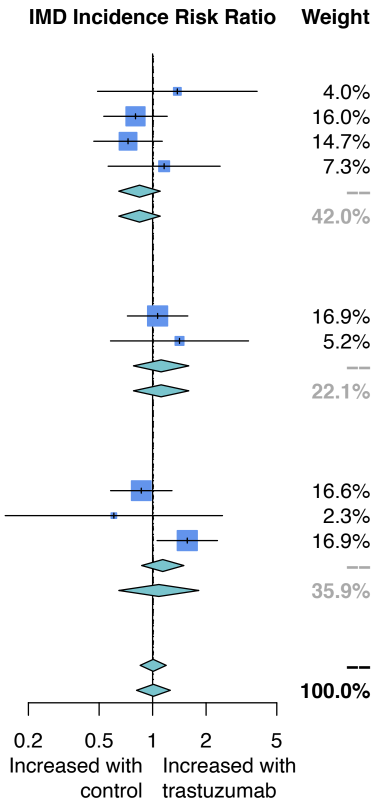

Supplementary Figure 3. Risk ratio of intracranial metastatic disease in patients receiving trastuzumab versus comparator for early stage HER2-positive breast cancer. Risk ratios were calculated from the proportion of patients in each study arm who developed intracranial metastatic disease over the study course. Studies here are stratified by comparator regimen: either chemotherapy, observation, or another HER2-targeted agent. The size of each box represents the weight of the contribution of each study to the weight of sample in the meta-analysis. The vertical dashed lines represent the points of summary for fixed and random effects models, and the diamonds represent 95% CI for the summary relative risks. Analyses were performed with the R programming language[26] and the R package meta.[27]

Study	Trastuzumab Events	Trastuzumab Total	Control Events	Control Total	RR	95%−CI	Weight
**Comparator = Anti−HER2**							
WJOG6110B/ELTOP	2	43	2	43	1.00	[0.15; 6.78]	1.7%
CEREBEL	12	250	8	251	1.51	[0.63; 3.62]	7.6%
NEfERT−T	41	237	20	242	2.09	[1.26; 3.46]	19.2%
LUX Breast−1	19	169	30	339	1.27	[0.74; 2.19]	17.0%
NCIC CTG MA.31	52	326	44	326	1.18	[0.82; 1.71]	29.4%
**Fixed effect model**	**126**	**1025**	**104**	**1201**	**1.40**	**[1.09; 1.80]**	**−−**
**Random effects model**					**1.42**	**[1.06; 1.90]**	**74.9%**
Heterogeneity: $I^2 = 0\%$, $\tau^2 = 0.0202$, $p = 0.48$							
**Comparator = Chemotherapy**							
Slamon 2001	42	234	21	235	2.01	[1.23; 3.28]	19.9%
GBG−BIG 03−05	8	78	5	78	1.60	[0.55; 4.68]	5.2%
**Fixed effect model**	**50**	**312**	**26**	**313**	**1.93**	**[1.24; 3.02]**	**−−**
**Random effects model**					**1.93**	**[1.24; 3.02]**	**25.1%**
Heterogeneity: $I^2 = 0\%$, $\tau^2 = 0$, $p = 0.71$							
**Fixed effect model**	**176**	**1337**	**130**	**1514**	**1.51**	**[1.22; 1.88]**	**−−**
**Random effects model**					**1.53**	**[1.19; 1.97]**	**100.0%**
Heterogeneity: $I^2 = 0\%$, $\tau^2 = 0.0211$, $p = 0.52$							

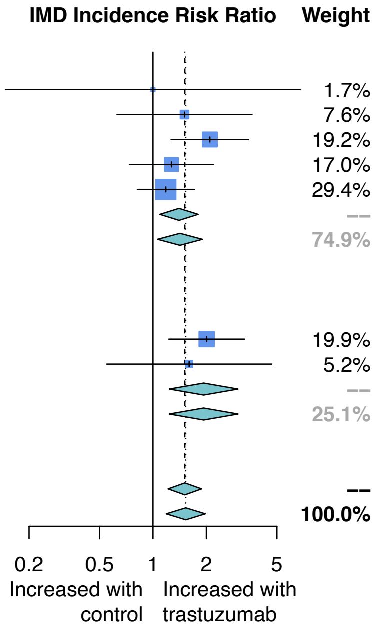

Supplementary Figure 4. Risk ratio of intracranial metastatic disease in patients receiving trastuzumab versus chemotherapy or another HER2-targeted agent for advanced stage HER2-positive breast cancer. Risk ratios were calculated from the proportion of patients in each study arm who developed intracranial metastatic disease over the study course. Studies here are stratified by comparator regimen: either chemotherapy or another HER2-targeted agent. The size of each box represents the weight of the contribution of each study to the weight of sample in the meta-analysis. The vertical dashed lines represent the points of summary for fixed and random effects models, and the diamonds represent 95% CI for the summary relative risks. Analyses were performed with the R programming language[26] and the R package meta.[27]

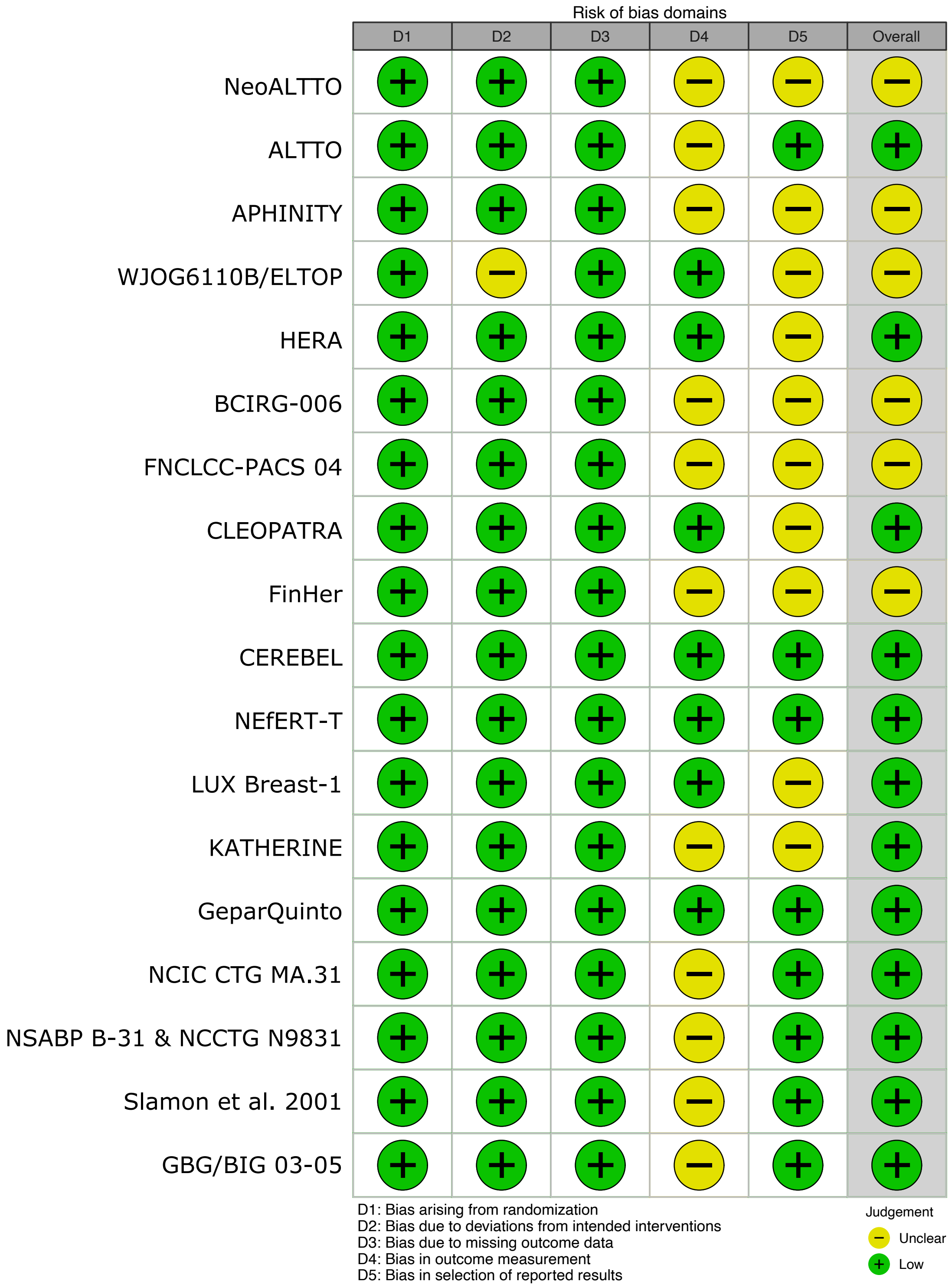

Supplementary Figure 5. Traffic light plot for risk of bias in included studies. The Cochrane Risk of Bias 2 tool (RoB 2) was employed to assess risk of bias in included studies.[1] The traffic light plot displays overall and domain-specific risk of bias for the included studies. Figure generated with the R programming language[26] and the R package robvis.[28]

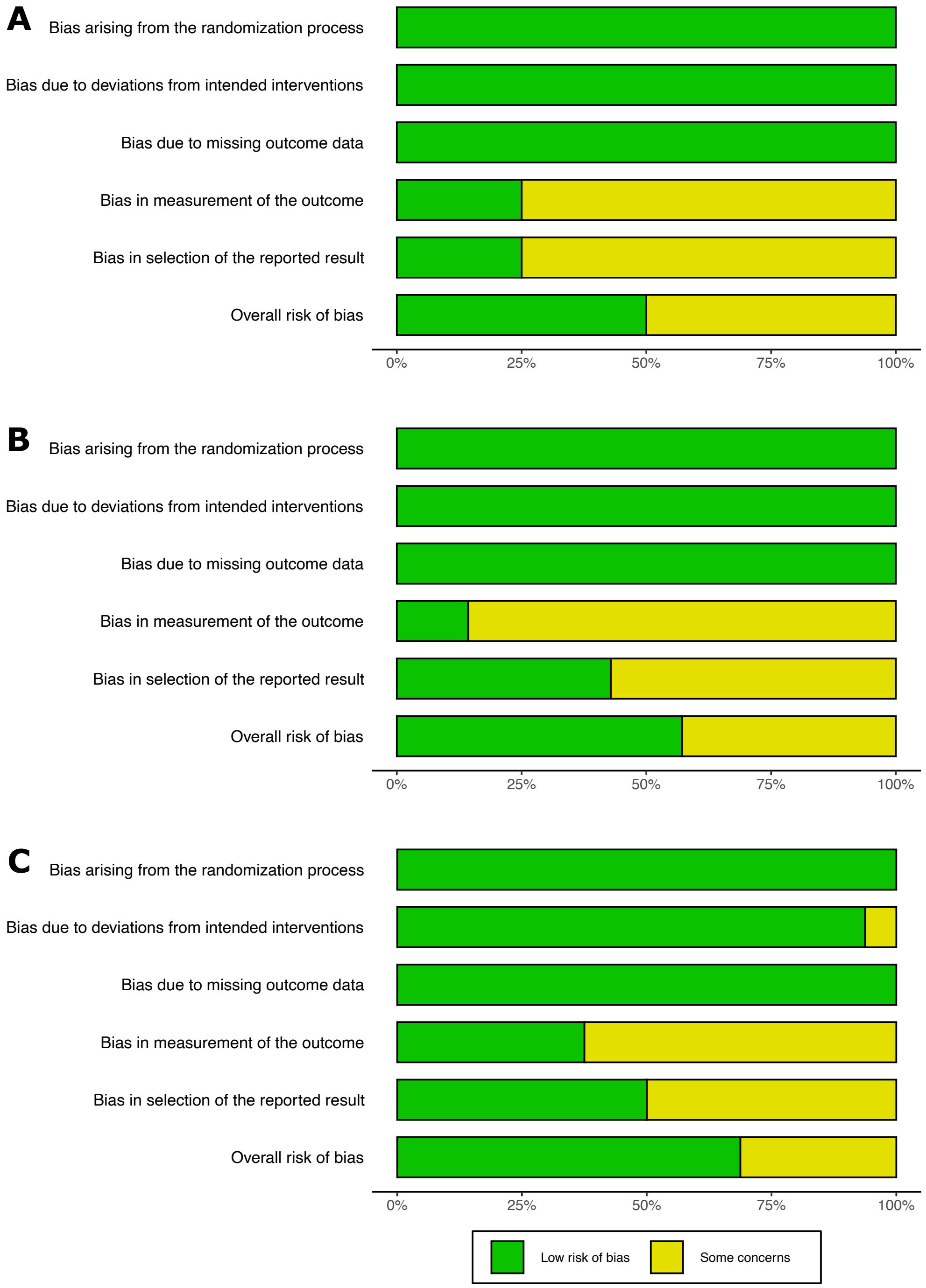

Supplementary Figure 6. Risk of bias summary plots. The Cochrane Risk of Bias 2 tool (RoB 2) was employed to assess risk of bias in included studies.[1] Figure generated with the R programming language[26] and the R package robvis.[28] A. Summary risk of bias in the comparison of intracranial metastatic disease incidence between dual- and single-agent HER2-targeted therapy regimens. B. Summary risk of bias in the comparison of intracranial metastatic disease incidence between trastuzumab and chemotherapy or observation. C. Summary risk of bias in the comparison of intracranial metastatic disease incidence between trastuzumab and chemotherapy or observation or another HER2-targeted agent.

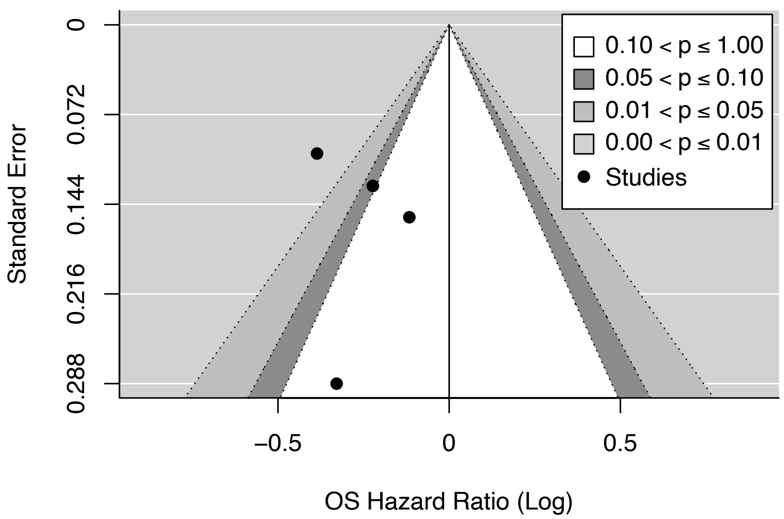

Supplementary Figure 7. Funnel plot for publication bias among studies reporting overall survival in dual- versus single-agent HER2-targeted therapy. Visual inspection of the funnel plot for clear asymmetry reveals potential publication bias, although too few studies are included here to power this assessment or Egger's test.

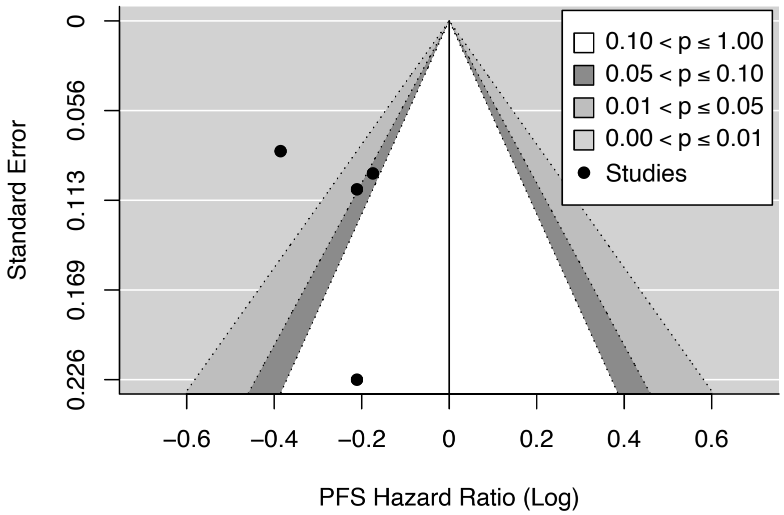

Supplementary Figure 8. Funnel plot for publication bias among studies reporting progression-free survival in dual- versus single-agent HER2-targeted therapy. Visual inspection of the funnel plot for clear asymmetry reveals potential publication bias, although too few studies are included here to power this assessment or Egger's test.

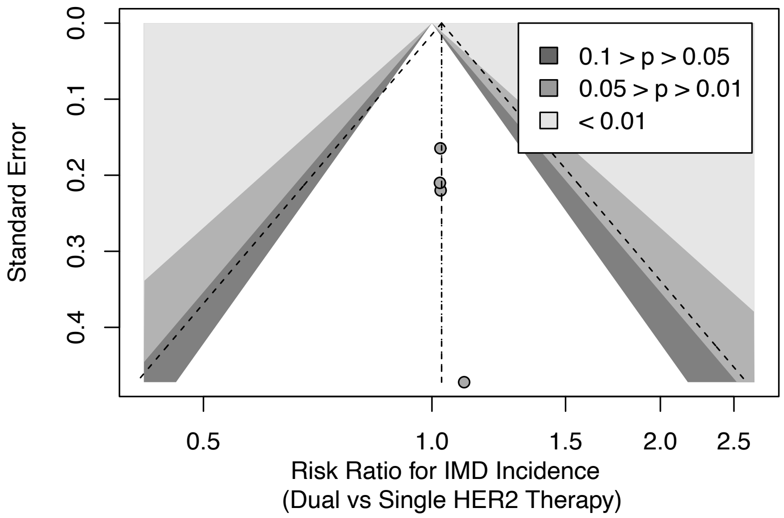

Supplementary Figure 9. Funnel plot for publication bias among studies reporting incidence of intracranial metastatic disease in dual- versus single-agent HER2-targeted therapy. Visual inspection of the funnel plot for clear asymmetry fails to reveal potential publication bias, although too few studies are included here to power this assessment or Egger's test.

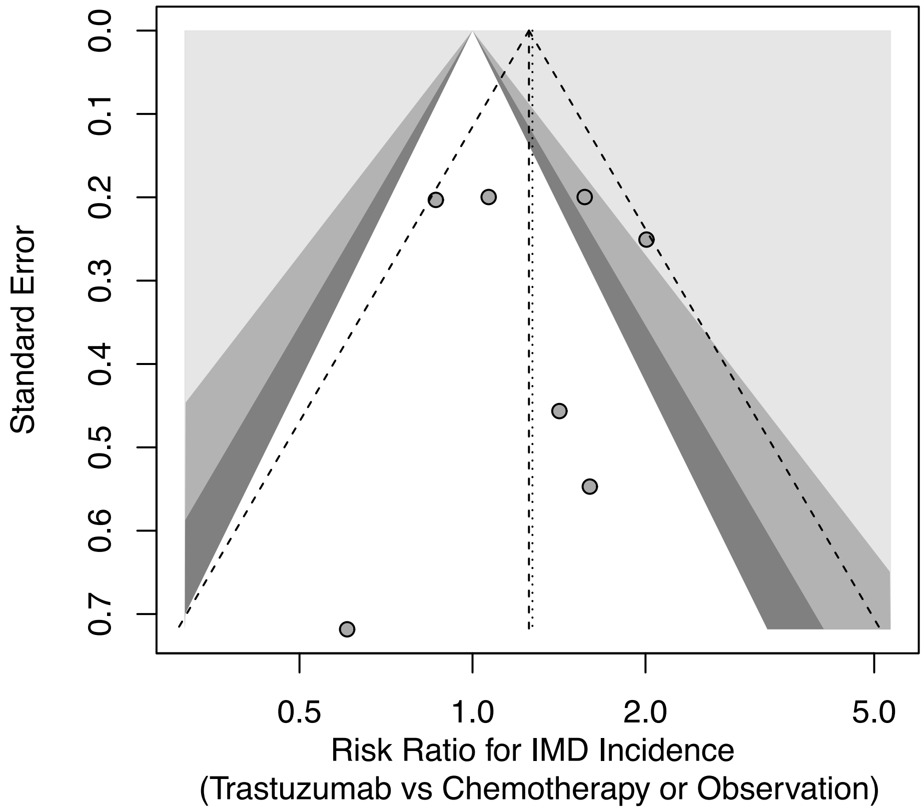

Supplementary Figure 10. Funnel plot for publication bias among studies reporting incidence of intracranial metastatic disease in trastuzumab versus chemotherapy or observation. Visual inspection of the funnel plot for clear asymmetry fails to reveal potential publication bias, although too few studies are included here to power this assessment or Egger's test.

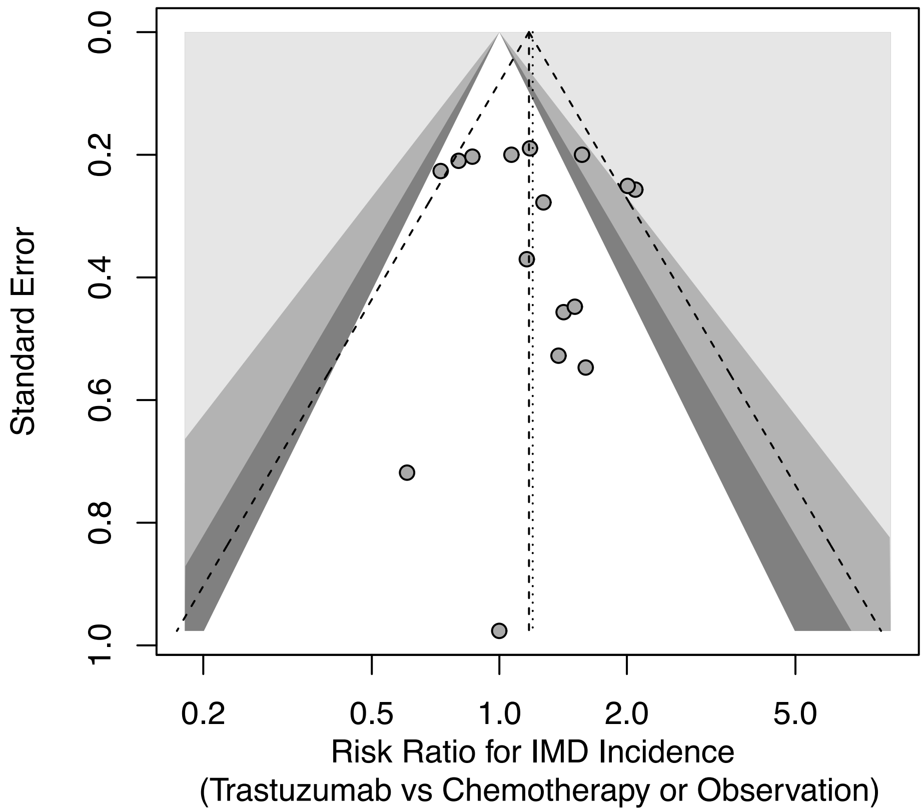

Supplementary Figure 11. Funnel plot for publication bias among studies reporting incidence of intracranial metastatic disease in trastuzumab versus chemotherapy, observation, or another HER2-targeted agent. Visual inspection of the funnel plot for clear asymmetry and Egger's test (p = .78) fail to reveal potential publication bias, although both methods are underpowered here.

Supplementary References:

1. Sterne JAC, Savović J, Page MJ, et al. RoB 2: a revised tool for assessing risk of bias in randomised trials. *BMJ.* 2019;366:l4898.
2. Michael Borenstein, L. V. Hedges, J. P. T. Higgins and H. R. Rothstein. Introduction to Meta-Analysis. © 2009 John Wiley & Sons, Ltd. ISBN: 978-0-470-05724-7. In.
3. Baselga J, Bradbury I, Eidtmann H, et al. Lapatinib with trastuzumab for HER2-positive early breast cancer (NeoALTTO): a randomised, open-label, multicentre, phase 3 trial. *Lancet.* 2012;379(9816):633-640.
4. Huober J, Holmes E, Baselga J, et al. Survival outcomes of the NeoALTTO study (BIG 1-06): updated results of a randomised multicenter phase III neoadjuvant clinical trial in patients with HER2-positive primary breast cancer. *Eur J Cancer.* 2019;118:169-177.
5. Piccart-Gebhart M, Holmes E, Baselga J, et al. Adjuvant Lapatinib and Trastuzumab for Early Human Epidermal Growth Factor Receptor 2-Positive Breast Cancer: Results From the Randomized Phase III Adjuvant Lapatinib and/or Trastuzumab Treatment Optimization Trial. *J Clin Oncol.* 2016;34(10):1034-1042.
6. von Minckwitz G, Procter M, de Azambuja E, et al. Adjuvant Pertuzumab and Trastuzumab in Early HER2-Positive Breast Cancer. *N Engl J Med.* 2017;377(2):122-131.
7. Takano T, Tsurutani J, Takahashi M, et al. A randomized phase II trial of trastuzumab plus capecitabine versus lapatinib plus capecitabine in patients with HER2-positive metastatic breast cancer previously treated with trastuzumab and taxanes: WJOG6110B/ELTOP. *Breast.* 2018;40:67-75.
8. Cameron D, Piccart-Gebhart MJ, Gelber RD, et al. 11 years' follow-up of trastuzumab after adjuvant chemotherapy in HER2-positive early breast cancer: final analysis of the HERceptin Adjuvant (HERA) trial. *Lancet.* 2017;389(10075):1195-1205.
9. Piccart-Gebhart MJ, Procter M, Leyland-Jones B, et al. Trastuzumab after adjuvant chemotherapy in HER2-positive breast cancer. *N Engl J Med.* 2005;353(16):1659-1672.
10. Romond EH, Perez EA, Bryant J, et al. Trastuzumab plus adjuvant chemotherapy for operable HER2-positive breast cancer. *N Engl J Med.* 2005;353(16):1673-1684.
11. Perez EA, Romond EH, Suman VJ, et al. Trastuzumab plus adjuvant chemotherapy for human epidermal growth factor receptor 2-positive breast cancer: planned joint analysis of overall survival from NSABP B-31 and NCCTG N9831. *J Clin Oncol.* 2014;32(33):3744-3752.
12. Slamon D, Eiermann W, Robert N, et al. Adjuvant trastuzumab in HER2-positive breast cancer. *N Engl J Med.* 2011;365(14):1273-1283.
13. Chan A, Spera G, MacHado A, et al. Central nervous system as first site of relapse in patients with HER2 positive early breast cancer treated in the BCIRG-006 trial. *Cancer Research.* 2019;Conference:2018 San Antonio Breast Cancer Symposium. United States. 2079 (2014 Supplement 2011) (no pagination).
14. Spielmann M, Roche H, Delozier T, et al. Trastuzumab for patients with axillary-node-positive breast cancer: results of the FNCLCC-PACS 04 trial. *J Clin Oncol.* 2009;27(36):6129-6134.
15. Swain SMBJMDIYHQCLLFaCJ. Incidence of central nervous system metastases in patients with HER2-positive metastatic breast cancer treated with pertuzumab, trastuzumab, and docetaxel: results from the randomized phase III study CLEOPATRA. *Annals of oncology : official journal of the european society for medical oncology.* 2014;25(6):1116-1121.
16. Swain SM, Baselga J, Kim SB, et al. Pertuzumab, trastuzumab, and docetaxel in HER2-positive metastatic breast cancer. *N Engl J Med.* 2015;372(8):724-734.
17. Joensuu H, Bono P, Kataja V, et al. Fluorouracil, epirubicin, and cyclophosphamide with either docetaxel or vinorelbine, with or without trastuzumab, as adjuvant treatments of breast cancer: final results of the FinHer Trial. *J Clin Oncol.* 2009;27(34):5685-5692.
18. Pivot X, Manikhas A, Zurawski B, et al. CEREBEL (EGF111438): A Phase III, Randomized, Open-Label Study of Lapatinib Plus Capecitabine Versus Trastuzumab Plus Capecitabine in

Patients With Human Epidermal Growth Factor Receptor 2-Positive Metastatic Breast Cancer. *Journal of Clinical Oncology.* 2015;33(14):1564-1573.

19. Awada A, Colomer R, Inoue K, et al. Neratinib Plus Paclitaxel vs Trastuzumab Plus Paclitaxel in Previously Untreated Metastatic ERBB2-Positive Breast Cancer: The NEfERT-T Randomized Clinical Trial. *JAMA Oncology.* 2016;2(12):1557-1564.
20. Harbeck N, Huang CS, Hurvitz S, et al. Afatinib plus vinorelbine versus trastuzumab plus vinorelbine in patients with HER2-overexpressing metastatic breast cancer who had progressed on one previous trastuzumab treatment (LUX-Breast 1): an open-label, randomised, phase 3 trial. *Lancet Oncol.* 2016;17(3):357-366.
21. von Minckwitz G, Huang CS, Mano MS, et al. Trastuzumab Emtansine for Residual Invasive HER2-Positive Breast Cancer. *N Engl J Med.* 2019;380(7):617-628.
22. Untch M, von Minckwitz G, Gerber B, et al. Survival Analysis After Neoadjuvant Chemotherapy With Trastuzumab or Lapatinib in Patients With Human Epidermal Growth Factor Receptor 2-Positive Breast Cancer in the GeparQuinto (G5) Study (GBG 44). *J Clin Oncol.* 2018;36(13):1308-1316.
23. Untch M, Loibl S, Bischoff J, et al. Lapatinib versus trastuzumab in combination with neoadjuvant anthracycline-taxane-based chemotherapy (GeparQuinto, GBG 44): a randomised phase 3 trial. *Lancet Oncol.* 2012;13(2):135-144.
24. Gelmon KA, Boyle FM, Kaufman B, et al. Lapatinib or trastuzumab plus taxane therapy for human epidermal growth factor receptor 2-positive advanced breast cancer: Final results of NCIC CTG MA.31. *Journal of Clinical Oncology.* 2015;33(14):1574-1583.
25. Slamon DJ, Leyland-Jones B, Shak S, et al. Use of chemotherapy plus a monoclonal antibody against HER2 for metastatic breast cancer that overexpresses HER2. *N Engl J Med.* 2001;344(11):783-792.
26. Team RC. R: a language and environment for statistical computing. 2019.
27. Schwarzer G. meta: an R package for meta-analysis. *R News.* 2007;7(3):40–45.
28. McGuinness LA (2019). “robvis: An R package and web application for visualising risk-of-bias assessments.” URL: https://github.com/mcguinlu/robvis .
